# Improving the Manchester Triage System for Pediatric Emergency Care: An International Multicenter Study

**DOI:** 10.1371/journal.pone.0083267

**Published:** 2014-01-15

**Authors:** Nienke Seiger, Mirjam van Veen, Helena Almeida, Ewout W. Steyerberg, Alfred H. J. van Meurs, Rita Carneiro, Claudio F. Alves, Ian Maconochie, Johan van der Lei, Henriëtte A. Moll

**Affiliations:** 1 Department of Pediatrics, Erasmus MC-Sophia Children's Hospital, Rotterdam, The Netherlands; 2 Department of Pediatrics, Hospital Fernando Fonseca, Amadora, Portugal; 3 Department Public Health, Erasmus MC, Rotterdam, Rotterdam, The Netherlands; 4 Department of Pediatrics, Haga Hospital- Juliana Children's Hospital, The Hague, The Netherlands; 5 Department of Pediatric Accident and Emergency, St. Mary's Hospital, Imperial College Healthcare NHS Trust, London, United Kingdom; 6 Department of Medical Informatics, Erasmus Medical Centre, Rotterdam, The Netherlands; University of Liverpool, United Kingdom

## Abstract

**Objectives:**

This multicenter study examines the performance of the Manchester Triage System (MTS) after changing discriminators, and with the addition use of abnormal vital sign in patients presenting to pediatric emergency departments (EDs).

**Design:**

International multicenter study

**Settings:**

EDs of two hospitals in The Netherlands (2006–2009), one in Portugal (November–December 2010), and one in UK (June–November 2010).

**Patients:**

Children (<16years) triaged with the MTS who presented at the ED.

**Methods:**

Changes to discriminators (MTS 1) and the value of including abnormal vital signs (MTS 2) were studied to test if this would decrease the number of incorrect assignment. Admission to hospital using the new MTS was compared with those in the original MTS. Likelihood ratios, diagnostic odds ratios (DORs), and c-statistics were calculated as measures for performance and compared with the original MTS. To calculate likelihood ratios and DORs, the MTS had to be dichotomized in low urgent and high urgent.

**Results:**

60,375 patients were included, of whom 13% were admitted. When MTS 1 was used, admission to hospital increased from 25% to 29% for MTS ‘very urgent’ patients and remained similar in lower MTS urgency levels. The diagnostic odds ratio improved from 4.8 (95%CI 4.5–5.1) to 6.2 (95%CI 5.9–6.6) and the c-statistic remained 0.74. MTS 2 did not improve the performance of the MTS.

**Conclusions:**

MTS 1 performed slightly better than the original MTS. The use of vital signs (MTS 2) did not improve the MTS performance.

## Introduction

The Manchester Triage System (MTS) is widely used in European emergency departments (EDs) and is based on 52 flowcharts, which incorporate the range of patients' presenting problems [1], [2]. Of these flowcharts, 49 flowcharts are suitable for children. The flowchart that best fits the child's presentation e.g. ‘abdominal pain in children’ or ‘abscesses and local infections’ is used by triage nurses to determine the urgency by which they should be seen by a physician.

Triage nurses work down this flowchart until one of the features (discriminators) is positive. This stops the triage process at that stage and the child needs to be seen within the allocated waiting time, corresponding to the triage category [1], [2]. The MTS triage categories are: 1) Immediate: immediate evaluation by a physician; 2) Very urgent: evaluation within 10 minutes; 3) Urgent: evaluation within one hour; 4) Standard: evaluation within two hours; and 5) Non-urgent: evaluation within four hours.

Misclassifying the MTS triage category for a patient can result in a longer waiting time (undertriage) or a shorter waiting time (overtriage). Undertriage, which can result in a patient's condition deteriorating whilst waiting to be seen, is more frequent in children with abnormal vital signs [3]. Overtriage may lead to delay in the assessment of truly unwell patients, particularly when there is a large number of patients (particularly of lesser urgency) waiting to be seen [4].

Earlier studies on the MTS in children established an independent reference standard for use as proxy for the true urgency of the patient to be seen [5], [6]. When comparing the original MTS with this reference standard, overtriage was more common in children older than 1 year presenting with medical problems, e.g. fever [6]. Modifying the MTS discriminators for these children improved MTS performance in terms of reducing overtriage without increasing undertriage in the Netherlands [7].

Modifications to the discriminators as presented in our previous study were made, and MTS version 1 was produced [7]. The addition of using vital signs (from an updated ranges of abnormal vital sign values taken from a recent systematic review) to the original MTS is termed MTS version 2 in this paper [8].

This international multicenter study evaluated the MTS performance of two modified versions of MTS for triaging pediatric patients at the ED.

## Methods

### Study design

Two adaptations of the MTS in three different countries were studied to see if they could improve overall performance. The adaptations were called ‘discriminator modifications, MTS version 1’ and with additional ‘vital sign modifications’, MTS version 2.

The study population was composed of children who had presented at the EDs of four European hospitals, triaged by the original first edition of the MTS.

In Portugal and the Netherlands the official Portuguese or Dutch translated versions of MTS were used [1].

Triage categories, according to MTS version 1 and MTS version 2, were produced by adapting the original MTS triage categories on the basis of allocated MTS flowchart, positive discriminator, age, or vital sign values. Thus, we retrospectively applied MTS 1 and MTS 2 on data that was prospectively collected by using the original MTS.

This study is part of an ongoing study on validation of the MTS [6], [7]; approved by the medical ethics committee of Erasmus MC. Requirement for informed consent was waived.

### Settings and selection of participants

Data collection included all children younger than 16 years in the following ED open for 24 hours a day.

Erasmus MC-Sophia Children's Hospital in Rotterdam, the Netherlands (May 2007–July 2009) is an inner-city university hospital with a multi-socio-economic and multi-ethnic population consisting of two million habitants. The pediatric ED receives approximately 9,000 children annually (44% self-referrals).

The Haga Hospital-Juliana Children's Hospital in The Hague, the Netherlands (August–December 2007) is a general teaching hospital in The Hague. The mixed adult-pediatric ED receives approximately 30,000 patient-visits per year, of whom 18,000 are pediatric patients (63% self-referrals). Since 1999, the ED has served as a trauma center with a catchment area of approximately one million habitants.

The St. Mary's Hospital in London, UK (June–November 2010) is a general teaching acute hospital with a catchment area of nearly 2 million habitants. It is the major trauma center for North West London. The pediatric emergency department sees 26,000 children a year (88% self-referrals).

The Fernando Fonseca Hospital in Lisbon, Portugal (November–December 2010) is an inner-city university hospital with a catchment area of 700,000 habitants. The pediatric ED receives nearly 60,000 children per year, predominantly self-referrals.

### Modifications of the MTS

#### MTS version 1

In the previous study, modifications to the discriminators for patient groups with high percentages of misclassification were evaluated in two hospitals in The Netherlands and improved the MTS performance [7]. In the MTS, there are general discriminators, e.g. hot child, which occur in most flowcharts and allocate to the same triage category, irrespective of their presenting problem. Our previous modifications were adjusted to this concept. The modifications (MTS version 1) are provided in table 1 [1], [2].

**Table 1:** Discriminator modifications for patients with high percentage of misclassification (MTS version 1).

Discriminator original MTS	Original MTS triage category	MTS version 1 discriminators	MTS version 1 triage categories	Cut-off level†
Hot child	Very urgent	Hot child <3 months	Very urgent	-
		Hot child >3 months	Urgent	86%
		Febrile child*	Very urgent	-
Persistent vomiting	Urgent	Persistent vomiting <3 months	Urgent	-
		Persistent vomiting >3 months	Standard	70%
Not feeding	Urgent	Not feeding <1 year	Urgent	-
		Not feeding >1 year	Standard	76%
Prolonged or uninterrupted crying	Urgent	Prolonged or uninterrupted crying <1 year	Urgent	-
		Prolonged or uninterrupted crying >1 year	Standard	100%
Scalp hematoma	Standard	Scalp hematoma <1 year	Standard	-
		Scalp hematoma >1 year	Non-urgent	66%
Unable to talk in sentences	Very urgent	Unable to talk in sentences	Urgent	75%
Wheeze	Standard	Wheeze	Urgent	53%‡

This discriminator is present in the neurological flowcharts: fits, irritable child, headache, crying baby, neck pain or behaving strangely.

Proportion of children that were allocated to a lower urgency triage category according to the independent reference standard.

Proportion of children that were allocated to a higher urgency triage category according to the independent reference standard.

#### MTS version 2

Vital signs were included as an additional discriminator to the original MTS, to produce MTS version 2 [3]. Heart rates and respiratory rates were considered abnormal if the first measured heart rate or respiratory rate was lower than the first percentile or higher than the 99^th^ percentile values published by Fleming et al. [8]. The cut-off levels are presented in the Table S1. The presence of abnormal heart or respiratory rates leads to a triage category of ‘very urgent’.

The discriminators ‘very low saturation’ and ‘low saturation’ were defined as peripheral oxygen saturation in air lower than 90% and lower than 95% respectively. If present, patients were triaged to MTS version 2 ‘very urgent’ and MTS version 2 ‘urgent’ triage categories. These discriminators were added to all flowcharts.

Although abnormal vital sign measurements were included in all flowcharts, vital sign recording was left to the nurse's discretion.

### Data collection

Data on MTS triage categories, the flowchart used for each patient, and the positive discriminator was collated from the computerized systems of MTS by trained triage nurses, experienced in both pediatric and emergency care [1]. The positive discriminator is the one that determines the MTS triage category.

Nurses recorded data on vital signs values, admission to hospital, and follow-up on structured ED templates. Heart rates, oxygen saturation, and temperature were collected in all four hospitals. Respiratory rates were not collected in the Fernando Fonseca Hospital in Lisbon, Portugal, because it was too time-consuming to measure respiratory rates routinely.

Triage categories, according to MTS version 1 and MTS version 2, were altered by adapting the original MTS triage categories on the basis of allocated MTS flowchart, positive discriminator, age, or vital sign values.

### Data analysis

MTS version 1 was initially derived for febrile children. To analyze the effect of this version, analysis on a febrile population was first performed. This population was defined as children who presented with a temperature higher than 38.4°C or had fever selected as triage discriminator. The next step was to analyze the performance of MTS version 1 in the total population to determine the overall improvement.

To assess the performances of MTS version 1 and MTS version 2, percentages of hospitalization of the original MTS categories were compared with the percentages of hospitalization of the new MTS categories. The MTS was deemed to have been improved if the hospitalization proportions increased in the higher urgency levels and/or lowered in the lower urgency levels.

Subsequently, the positive and negative likelihood ratios, diagnostic odds ratios (DORs), c-statistics (area under the receiver operator-curve), and R^2^ were calculated as measures for performance. The DOR is a measure of test performance that combines the sensitivity and specificity (sensitivity/1-sensitivity)/(1-specificity/specificity) [9]. To calculate the DOR and likelihood ratios the triage categories were ordered into ‘high urgent’ (MTS levels ‘immediate’ and ‘very urgent’) and ‘low urgent’ (MTS levels ‘urgent’, ‘standard’, and ‘non-urgent’). The c-statistics of the original MTS and the adapted versions of the MTS were compared by using the nonparametric approach of DeLong et al. [10] A p-value of less than 0.05 was considered significant.

Missing vital signs were imputed using a multiple imputation model including age, vital sign values, MTS category, presenting problem, and follow-up. This imputation process resulted in ten databases on which statistical analysis were performed and pooled for a final result [11].

Imputation was performed by using Design and Hmisc (AregImpute function) in R packages version 2.15.2. Statistical Packages for the Social Sciences (SPSS) version 20.0 (Chicago, IL) was used for the statistical analysis. The final database for the analysis on MTS 1 and 2 are attached as a supplementary file to the article. (Data S1)

## Results

### Study population

In total 64,653 children had presented to the EDs. Ninety-four percent (N = 60,800) were triaged using the MTS. Data on discharge or hospitalization were available for 60,735 patients. In total, 6,895 (11%) patients were admitted to hospital, of whom 29 died in the ED. Patients' characteristics are provided in table 2.

**Table 2:** Patients' characteristics per hospital.

	Sophia Children's Hospital		Juliana Children's Hospital		St. Mary's Hospital		Fernando Fonseca Hospital		Total population	
	N_total_ = 14,507 (%)		N_total_ = 5,299 (%)		N_total_ = 29,524 (%)		N_total_ = 11,405 (%)		N_total_ = 60,735 (%)	
**Age (years)**										
Median (IQR)	4.0	(1.3 to 9.2)	2.7	(0.9 to 6.7)	3.8	(1.5 to 8.5)	4.5	(1.7 to 9.0)	3.8	(1.4 to 8.6)
**Presenting problem**										
Trauma	2,856	(20)	873	(17)	6,171	(32)	983	(9)	10,883	(18)
Dyspnea	1,428	(10)	1,033	(20)	3,520	(12)	2,484	(22)	8,465	(14)
Gastro-intestinal	2,042	(14)	819	(16)	5,075	(17)	1,665	(15)	9,601	(16)
Ear, nose, throat	362	(3)	215	(4)	1,805	(6)	1,830	(16)	4,212	(7)
Wounds	1,111	(8)	349	(7)	1,405	(5)	242	(2)	3,107	(5)
Neurological	1,115	(8)	208	(4)	1,448	(5)	409	(4)	3,180	(5)
Fever without source	845	(6)	493	(9)	928	(3)	54	(1)	2,320	(4)
Rash	358	(3)	164	(3)	2,671	(9)	579	(5)	3,772	(6)
Urinary tract problems	335	(2)	102	(2)	292	(1)	247	(2)	976	(2)
Local infection/abscess	232	(2)	91	(2)	186	(1)	183	(2)	692	(1)
Other problems	3,823	(26)	952	(18)	6,023	(20)	2,729	(24)	13,527	(22)
**Original MTS triage category**										
Immediate	329	(2)	102	(2)	297	(1)	51	(0)	779	(1)
Very urgent	2,855	(20)	942	(18)	3,537	(12)	2,288	(20)	9,622	(16)
Urgent	6,253	(43)	1,283	(24)	4,338	(15)	2,277	(20)	14,151	(23)
Standard/non-urgent	5,070	(35)	2,972	(56)	21,352	(72)	6,789	(60)	36,183	(60)
**Follow-up**										
No follow-up	5,572	(38)	3,104	(59)	15,383	(52)	6,573	(58)	30,632	(50)
Outpatient clinic/GP	5,055	(35)	1,192	(22)	8,387	(28)	3,999	(35)	18,633	(31)
Hospital admission	2,720	(19)	755	(14)	2,866	(10)	554	(5)	6,895	(11)
Other follow-up	1,160	(8)	248	(5)	2,888	(10)	279	(2)	4,575	(8)

### MTS version 1 in the febrile population

Among the 6,836 febrile children eligible for analyses, 19% (N = 1,302) were hospitalized.

One percent (N = 80) were triaged ‘immediate’; 63% (N = 4,310) ‘very urgent’; 18% (N = 1,259) ‘urgent’; 17% (N = 1,184) ‘standard’; and less than 1% (N = 3) ‘non-urgent’.

In total, 3,162 (46%) children were reclassified to either a higher or lower triage category.

The proportions of hospitalization increased in the MTS ‘very urgent’ level from 20% to 37% and decreased in the MTS ‘urgent’ level from 23% to 16%, while there were no differences in the other MTS urgency levels.

The positive likelihood ratio increased from 1.1 (95%CI 1.1–1.2) to 2.6 (95%CI 2.4–2.9) and the negative likelihood ratio decreased from 0.80 (95%CI 0.73–0.87) to 0.71 (95%CI 0.68–0.74). The DOR improved from 1.4 (95%CI 1.2–1.6) to 3.7 (95%CI 3.3–4.7), the R^2^ improved from 0.05 to 0.10 and the c-statistic increased significantly from 0.56 (95%CI 0.55–0.58) to 0.66 (95%CI 0.64–0.67, p-value<0.001). (Table 3)

**Table 3:** Performance of the original MTS, MTS version 1, and MTS version 2.

Data	MTS edition	Positive likelihood	Negative likelihood	Diagnostic odds ratio†	c-statistic	R^2^
**MTS version 1**						
Fever (N = 6,836)						
	Original MTS	1.1 (1.1 to 1.2)	0.80 (0.73 to 0.87)	1.4 (1.2 to 1.6)	0.56 (0.55 to 0.58)	0.05
	MTS version 1	2.6 (2.4 to 2.9)	0.71 (0.68 to 0.74)	3.7 (3.3 to 4.3)	0.66 (0.64 to 0.67)	0.10
Total population (N = 60,735)						
	Original MTS	3.2 (3.0 to 3.3)	0.66 (0.64 to 0.67)	4.8 (4.6 to 5.1)	0.74 (0.73 to 0.74)	0.17
	MTS version 1	4.3 (4.1 to 4.4)	0.69 (0.67 to 0.70)	6.2 (5.9 to 6.6)	0.74 (0.74 to 0.75)	0.18
**MTS version 2**						
Heart rate (N = 60,735)						
	Original MTS	3.2 (3.0 to 3.3)	0.66 (0.64 to 0.67)	4.8 (4.6 to 5.1)	0.74 (0.73 to 0.74)	0.17
	MTS version 2	2.2 (2.1 to 2.2)	0.74 (0.74 to 0.75)	3.6 (3.4 to 3.8)	0.71 (0.71 to 0.72)	0.16
Respiratory rate (N = 49,330)*						
	Original MTS	3.5 (3.3 to 3.6)	0.65 (0.64 to 0.66)	5.3 (5.0 to 5.7)	0.74 (0.74 to 0.75)	0.19
	MTS version 2	2.4 (2.3 to 2.5)	0.60 (0.58 to 0.61)	4.0 (3.8 to 4.2)	0.73 (0.72 to 0.73)	0.16
Oxygen saturation (N = 60,735)						
	Original MTS	3.2 (3.0 to 3.3)	0.66 (0.64 to 0.67)	4.8 (4.6 to 5.1)	0.74 (0.73 to 0.74)	0.17
	MTS version 2	3.2 (3.1 to 3.3)	0.65 (0.64 to 0.66)	4.9 (4.6 to 5.1)	0.74 (0.74 to 0.75)	0.18

Respiratory rates were measured in the Sophia Children's Hospital, Juliana Children's Hospital and St. Mary's Hospital.

Diagnostic odds ratio = DOR = (sensitivity/1-sensitivity)/(1-specificity/specificity).

### MTS version 1 in the total population

Using the MTS version 1 in the total population (N = 60,735), 4,526 (7%) children were reclassified of whom 3,991 were allocated to a lower urgency level. Hospitalization increased in the MTS ‘very urgent’ triage category from 25% to 29%, while they remained similar in the other MTS urgency levels. Table 4 shows the total reclassification.

**Table 4:** Reclassification table: The original MTS compared with MTS version 1.

	MTS version 1					
Original MTS	Immediate	Very urgent	Urgent	Standard	Non urgent	Total
Immediate	779	0	0	0	0	779
Hospitalization (%)	552 (67.0)					552 (67.0)
Very urgent	0	6,562	3,061	0	0	9,623
Hospitalization (%)		2,068 (31.5)	402 (13.1)			2,470 (25.7)
Urgent		0	13,221	930	0	14,151
Hospitalization (%)			2,211 (16.7)	147 (15.8)		2,358 (16.7)
Standard	0	0	535	35,254	119	35,908
Hospitalization (%)			89 (16.6)	1,449 (4.1)	1 (0.8)	1,539 (4.3)
Non urgent	0	0	0	0	274	274
Hospitalization (%)					6 (2.2)	6 (2.2)
Total	779	6,562	16,817	36,184	393	60,735
Hospitalization (%)	552 (67.0)	2,068 (31.5)	2,702 (16.0)	1,596 (4.4)	7 (1.8)	6,895 (11.4)

The overall positive likelihood ratio of the MTS improved significantly from 3.2 (95%CI 3.0–3.3) to 4.3 (95%CI 4.1–4.4). The DOR increased from 4.8 (95%CI 4.6–5.1) to 6.2 (95%CI 5.9–6.6), the R^2^ changed from 0.17 to 0.18 and the c-statistic remained 0.74. (Table 3)

**Figure 1:**
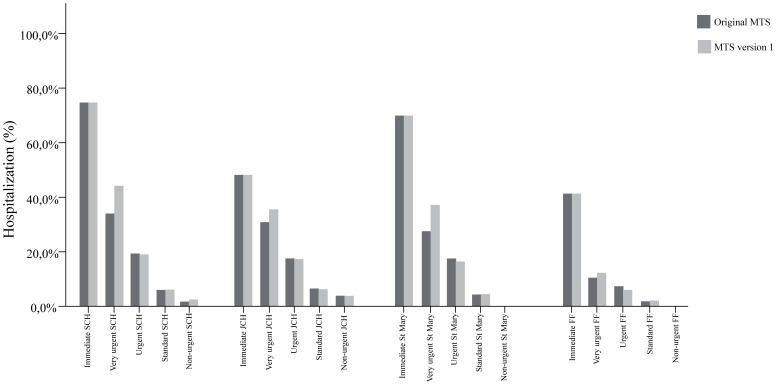
Percentages of hospitalization per urgency level in Sophia Children's Hospital (SCH), Juliana's Children's Hospital (JCH), St Mary Hospital, and in Fernando Fonseca Hospital (FF).

If percentages of hospitalization were compared for the three hospitals separately, similar trends in percentages of hospitalization were found. (Figure 1) The likelihood ratios, DORs, R^2^ and c-statistics for the separate hospitals are shown in table 5. In all hospitals, the modifications showed the same results although the results of the Fernando Fonseca hospital were not statistically significant.

**Table 5:** Performance of the original MTS, MTS version 1, and MTS version 2 per hospital.

Data	MTS edition	Positive likelihood	Negative likelihood	DOR	c-statistic	R^2^
*Sophia Children's Hospital (N = 14,507)*						
	Original MTS	2.7 (2.5–2.8)	0.67 (0.64–0.69)	4.0 (3.7–4.4)	0.71 (0.70–0.72)	0.17
	MTS version 1	4.1 (3.8–4.4)	0.68 (0.66–0.70)	6.1 (5.5–6.7)	0.72 (0.71–0.73)	0.19
	MTS version 2 (heart rate)	2.0 (1.9–2.2)	0.60 (0.55–0.65)	3.4 (2.9–4.0)	0.70 (0.69–0.71)	0.15
	MTS version 2 (respiratory rate)	1.9 (1.8–2.0)	0.63 (0.60–0.66)	3.0 (2.8–3.3)	0.69 (0.67–0.71)	0.14
	MTS version 2 (oxygen saturation)	2.7 (2.5–2.8)	0.66 (0.64–0.68)	4.0 (3.7–4.4)	0.71 (0.70–0.72)	0.17
*Juliana Children's Hospital (N = 5,299)*						
	Original MTS	2.9 (2.6–3.2)	0.65 (0.61–0.70)	4.4 (3.8–5.2)	0.71 (0.69–0.73)	0.14
	MTS version 1	3.6 (3.2–4.0)	0.68 (0.64–0.72)	5.2 (4.4–6.2)	0.72 (0.70–0.74)	0.15
	MTS version 2 (heart rate)	2.9 (2.6–3.2)	0.65 (0.61–0.70)	4.4 (3.8–5.2)	0.69 (0.67–0.71)	0.11
	MTS version 2 (respiratory rate)	2.1 (2.0–2.2)	0.57 (0.52–0.62)	3.7 (3.2–4.4)	0.70 (0.69–0.73)	0.12
	MTS version 2 (oxygen saturation)	3.0 (2.7–3.3)	0.64 (0.60–0.68)	4.6 (3.9–5.5)	0.72 (0.70–0.74)	0.15
*St. Mary's Hospital (N = 29,524)*						
	Original MTS	4.1 (3.9–4.4)	0.65 (0.63–0.67)	6.3 (5.8–6.9)	0.74 (0.73–0.75)	0.18
	MTS version 1	6.5 (6.0–7.0)	0.69 (0.67–0.71)	9.4 (8.6–10.3)	0.74 (0.73–0.75)	0.20
	MTS version 2 (heart rate)	4.3 (4.0–4.7)	0.78 (0.77–0.78)	4.3 (4.0–4.7)	0.72 (0.71–0.73)	0.15
	MTS version 2 (respiratory rate)	2.7 (2.6–2.9)	0.61 (0.59–0.63)	4.5 (4.2–4.9)	0.72 (0.71–0.73)	0.15
	MTS version 2 (oxygen saturation)	4.1 (4.0–4.3)	0.65 (0.63–0.67)	6.4 (5.9–6.9)	0.74 (0.73–0.75)	0.18
*Fernando Fonseca Hospital (N = 11,405)*						
	Original MTS	2.5 (2.2–2.7)	0.65 (0.60–0.71)	3.8 (3.2–4.5)	0.72 (0.69–0.74)	0.10
	MTS version 1	2.9 (2.7–3.2)	0.64 (0.59–0.69)	4.6 (3.9–5.5)	0.71 (0.69–0.74)	0.10
	MTS version 2 (heart rate)	1.8 (1.6–1.9)	0.64 (0.58–0.70)	2.8 (2.3–3.3)	0.68 (0.65–0.70)	0.08
	MTS version 2 (oxygen saturation)	2.5 (2.2–2.7)	0.65 (0.60–0.70)	3.8 (3.2–4.5)	0.71 (0.69–0.74)	0.11

### MTS version 2

Heart rates were measured in 52% (N = 31,707) of the total population (N = 60,735); respiratory rates were measured in 48% (N = 23,513) of patients who visited the hospitals in the Netherlands and the UK (N = 49,330 patients); and oxygen saturation was measured in 46% (N = 28,066) of patients.

Heart rate modifications reclassified 7,298 patients (12%) to the higher MTS ‘very urgent’ triage category when compared to the original MTS. Eleven percent (N = 829) of the reclassified patients were hospitalized.

Respiratory rate modification reclassified 4,949 patients (10%). Thirteen percent (N = 666) of these were hospitalized.

Oxygen saturation modifications reclassified 130 patients (<1%) to the MTS ‘very urgent’ triage category (of whom 47 (36%) were hospitalized) and 220 patients (<1%) to the MTS ‘urgent’ triage category (of whom 57 (26%) were hospitalized).

The performance of MTS version 2 did not improve irrespective of the use of heart rate and respiratory rate. (Table 3) The addition of oxygen saturation slightly changed the R^2^ and the c-statistics; however there were no statistically significant improvements of likelihood ratios and diagnostic odds ratios.

## Discussion

This international multicenter study showed that discriminator modifications of the MTS (MTS version 1) improved the performance of the MTS when hospitalization was used as surrogate marker for urgency. Moreover, MTS version 1 did not increase the hospitalization percentages in the lowest urgency levels. Vital signs modifications (MTS version 2) did not improve the performance of MTS.

### MTS version 1

MTS version 1, reclassified only 7% of the total population. This seems a small number and therefore the impact on the total performance may be minimal. However, 88% of those patients were reclassified to a lower urgency level and therefore influences on workflow and pressure could be substantial, as maximum waiting times are extended to at least 50 minutes. The modifications were initially developed for children with infectious presenting symptoms. When analyses were performed on the febrile population 46% patients were reclassified and both DOR as c-statistic increased significantly.

In the pediatric Canadian Triage and Acuity Scale (CTAS) and the Emergency Severity Index (ESI), modifications for febrile children were implemented as well [12], [13]. In the pediatric CTAS, waiting times for febrile children older than three months without signs of ‘toxicity’, toxicity meaning unexplained crying before examination, difficulty awakening, or poor response to the physical evaluation, were extended from 15 minutes to 30 minutes and waiting times for febrile children older than three years were extended from 30 minutes to 60 minutes [13].

The modifications of the ESI were on the basis of the guideline “Clinical Policy for Children Younger than 3 Years Presenting to the Emergency Department with Fever” published by The American College of Emergency Physicians [14]. Children older than three months with a temperature higher than 39.0°C could be down-triaged at least one triage category [12]. Modifications of the ESI were based on literature review, but the impact of these specific modifications for children was, to our knowledge, not evaluated after implementation.

The changes in MTS version 1 are similar to the modifications implemented in other triage systems and are evaluated in four different settings in three different countries. The implementations improved the MTS ability to distinguish the degree of urgency and therefore we recommend incorporation of the modifications in the next version of the MTS.

### MTS version 2

Before the introduction of formal triage systems, vital signs were often used as a decision making tool to determine how quickly a patient should be seen [15]. Since the introduction of five-level triage scales, the role of vital signs as an urgency marker still exists, but is not predominant as decision making tool anymore.

In the Australasian Triage Scale (ATS), the pediatric CTAS, and ESI, vital signs are measured in less urgent triage categories to upgrade patients with abnormal vital signs [13], [15], [16].

In the original MTS, vital signs are only incorporated in specific flowcharts and therefore only measured in patients presenting with specific presenting symptoms [1], [2]. Vital signs have been thought to be an essential component of pediatric triage [17]. Our previous study [3] suggested that vital sign measurement might help reduce undertriage rates. Given these two factors, the values for normal and abnormal vital signs were added to all flowcharts. In contrast to our expectations, our study showed that MTS version 2 did not benefit from introducing vital signs measurements.

These results can partially be explained by knowledge of abnormal vital sign values at triage assessment. Studies have shown that in six to eight percent of patients, triage decisions were affected by knowledge of vital signs [18], [19]. These percentages were 11.4% for younger children [19]. However, none of these studies have analyzed the correctness of changing the triage decision. Moreover, no cut-off levels for abnormal vital signs were given to these nurses [19] and therefore change in triage decision in these studies were not based on evidence based cut-off values, but on the interpretation by the nurse.

In our study, the cut-off levels of abnormal vital signs were on the basis of evidence-based reference ranges [8]. Cut-off level for abnormal vital sign were the first and 99^th^ percentile of these reference ranges, because these extreme levels are associated with the most severely ill population.

### Strength and limitations

The modifications were evaluated in four different settings. Although the included time period varied per hospital and therefore case-mix could have affected our results, the modifications showed the same results for MTS version 1 and MTS version 2 in all hospitals. Moreover, the seasonal influences upon the evaluation of the triage decisions were not statistically significant in the hospitals in which data was collected for at least one year. (Data not shown) This indicates that modifications of the MTS can be generalized to other developed countries regardless of health care system or MTS translation.

In this study, the modifications of the MTS were not implemented in the triage process itself and therefore the modifications were not evaluated in practice. As the modifications to the MTS are small and simple, we expect comparable performance in practice.

In earlier studies, we argued that it is preferred to evaluate triage systems with an independent reference standard [5]–[7], [20]. However, this reference standard is based on many different items which were not available in the various settings. Since MTS version 2 has incorporated vital signs to the MTS, a reference standard including vital signs is not independent of MTS version 2 and therefore not suitable. For these reasons, hospitalization was used as a surrogate marker for severity. Criteria for hospitalization were abnormal or threatened vital signs, requirements of intravenous medication or fluids, failure to ingest medication (e.g., need for a nasogastric tube), and requirements for surgery. We are aware that hospitalization may not always mean the patient must be seen within 10 minutes or that discharged patients can wait for at least one hour [21]. For example, patients with respiratory distress stabilized after receiving a nebulizer should be seen within 10 minutes after arrival, but the patient may be subsequently discharged. Despite this limitation, the marker of hospitalization is associated with patients being classified as ‘urgent’ in other studies on pediatric triage [22]–[24].

Vital signs were only measured in 50% of patients. Literature showed that there is a correlation between triage nurse measurement of vital signs and the severity of the presenting illness and thus missing at random on x (vital signs) and y (hospitalization) [25], [26]. A valid method to deal with missing at random is a multiple imputation model that replaces the missing value by a value that is drawn from an estimate of the distribution of the variable [11], [27].

### Conclusions

Discriminator modifications (MTS version 1) improve the performance of the MTS in this broad validation study in different international EDs.

We recommend implementing these modifications in the next version of the MTS. The addition of vital signs to the MTS (MTS version 2) did not improve triage classifications.

## Supporting Information

Table S1 **Reference values for normal heart rates and respiratory rates by Fleming et al.** [**8**] **.**
(DOC)

Data S1 **Database main analysis PLOS one.xls.**
(XLS)
