# Supplementary material for: Improving the Manchester Triage System for Pediatric Emergency Care: An International Multicenter Study
Source: PLoS One. 2014 Jan 15;9(1):e83267. doi: 10.1371/journal.pone.0083267 (PMC3893080; doi:10.1371/journal.pone.0083267)
Supplement: Table S1 — Reference values for normal heart rates and respiratory rates by Fleming et al. [8] . (DOC) [file pone.0083267.s001.doc]

| **Age range (months/years)** | **Heart rate**  **(beats per minute)** | **Respiratory rate**  **(breaths per minute)** |
| --- | --- | --- |
| 0 to 3 months | 107 to 181 | 25 to 66 |
| 3 to 6 months | 104 to 175 | 24 to 64 |
| 6 to 9 months | 98 to 168 | 23 to 61 |
| 9 to 12 months | 93 to 161 | 22 to 58 |
| 12 to 18 months | 88 to 156 | 21 to 53 |
| 18 to 24 months | 82 to 149 | 19 to 46 |
| 2 to 3 years | 76 to 142 | 18 to 38 |
| 3 to 4 years | 70 to 136 | 17 to 33 |
| 4 to 6 years | 65 to 131 | 17 to 29 |
| 6 to 8 years | 59 to 123 | 16 to 27 |
| 8 to 12 years | 52 to 115 | 14 to 25 |
| 12 to 15 years | 47 to 108 | 12 to 23 |
| 15 to 16 years | 43 to 104 | 11 to 22 |
